# Political borders impact associations between habitat suitability predictions and resource availability

**DOI:** 10.1007/s10980-020-01103-8

**Published:** 2020-09-04

**Authors:** Matthias Tschumi, Patrick Scherler, Julien Fattebert, Beat Naef-Daenzer, Martin U. Grüebler

**Affiliations:** 1 Swiss Ornithological Institute, Seerose 1, 6204 Sempach, Switzerland; 2 Institute of Evolutionary Biology and Environmental Studies, University of Zurich, Winterthurerstrasse 190, 8057 Zurich, Switzerland; 3 School of Life Sciences, University of KwaZulu-Natal, Durban, 4000 South Africa

**Keywords:** Agri-environment schemes, Birds, Ground-truthing, Habitat suitability models, Land use, Landscape simplification

## Abstract

**Context:**

By linking species of conservation concern to their abiotic and biotic requirements, habitat suitability models (HSM) can assist targeted conservation measures. Yet, conservation measures may fail if HSM are unable to predict crucial resources. HSM are typically developed using remotely sensed land-cover classification data but not information on resources per se.

**Objectives:**

While a certain land-cover class may correlate with crucial resources in the area of calibration, political boundaries can abruptly alter these associations. We investigate this potential discrepancy in a well-known study system highly relevant for farmland bird conservation.

**Methods:**

We compared land cover, land-use intensity and resource availability between plots of highest habitat suitability for little owls (*Athene noctua*) among two neighbouring, but politically separated areas (i.e. south-western Germany vs. northern Switzerland).

**Results:**

Land cover and land-use richness did not differ between German and Swiss plots. Yet there were marked differences in terms of land-use intensity and the availability of resources. Land-use intensity was significantly higher and resource availability lower in Swiss compared to German plots.

**Conclusions:**

While accounting well for remotely sensed data such as land cover, HSM may fail to predict land-use intensity and resources across borders. The relationship between geodata used as proxies and ecologically relevant resources may differ according to history, policies and socio-cultural context, constraining the viability of HSM across political borders. This study emphasises the need for fine-scale resource assessments complementing landscape-scale suitability models. Conservation measures need to consider the availability of crucial resources and their socio-economic moderators to be effective.

**Electronic supplementary material:**

The online version of this article (10.1007/s10980-020-01103-8) contains supplementary material, which is available to authorized users.

## Introduction

Habitat suitability models (HSM) are important tools for designing evidence-based conservation measures (Hirzel and Le Lay 2008). Based on environmental data and species occurrence records, HSM allow predicting the potential distribution of species over large spatial scales (Boyce and McDonald 1999). The resulting habitat suitability maps assist species-specific conservation measures to be prioritized spatially (and temporally) to where they are most likely to be successful. Yet, despite laborious conservation actions, suitable areas often remain unoccupied (e.g. Marcer et al. 2013; Fattebert et al. 2018). As an example, the provisioning of 230 little owl nest boxes in areas of high predicted habitat suitability in central Switzerland has not resulted in a population recovery so far (Fattebert et al. 2018; Grüebler and Tschumi 2019). There are three major explanations for this: (A) There are limiting demographic factors (e.g. lacking source population; Schaub et al. 2006), (B) there are unfavourable landscape aspects (e.g. low functional connectivity; Hauenstein et al. 2019), or (C) the habitat does not contain the required resources to sustain the individual requirements, although the predicted suitability is sufficient (Brambilla et al. 2009).

HSM are often generated based on large-scale land-cover classification maps and topographic data derived from administrative databases or remote sensing (Boyce and McDonald 1999; Rushton et al. 2004). By linking this information to species occurrence records, HSM derive proxies for resources that are crucial for its occurrence (Boyce and McDonald 1999; Rushton et al. 2004). The underlying assumption is, that the disproportional use of certain habitat features is related to the availability of crucial resources and consequently to the species’ fitness (Morris 2003; Hirzel and Le Lay 2008). HSM thus statistically compare selected to available habitat features and subsequently predict the probability of species occurrence to the landscape (Hirzel and Le Lay 2008). Because the resources themselves are often fine scaled, they are difficult to be captured by administrative databases or remote sensing and HSM thus rarely account for the availability of critical resources per se (Rushton et al. 2004; Brambilla et al. 2009). HSM thus inevitably assume that proxies based on geodata are consistent in their correlation to resources across the full spatial extent of concern.

However, resource availability can change within small distances across political boundaries (Schmid and Pasinelli 2002; Arrondo et al. 2018). Differences in history, policies and socio-cultural context may result in different land-use intensity and structural configuration within the same land cover class and thus in different resource availability (Batáry et al. 2017). Although boundaries leading to discrete changes can also occur within political entities, political borders are likely to modify multiple aspects simultaneously (Batáry et al. 2017). In farmland, historical changes in mechanisation, anthropogenic inputs and structural transformations were fundamental drivers of species declines (Robinson and Sutherland 2002). Although overall developments were similar across Europe, there are substantial regional differences in intensification outcomes (e.g. Wretenberg et al. 2006; Batáry et al. 2017). To mitigate negative effects of agricultural intensification, agro-environmental policy has defined laws and regulations, as well cross-compliance mechanisms for farmers to receive subsidies conditional to compliance with environmental standards (Herzog et al. 2017). In central Europe, many ecological management guidelines and amenities are defined in comprehensive agri-environment schemes (AES; Batáry et al. 2015). While most AES offer support for semi-natural habitats, crop diversification or reduced-input farming, specific measures differ among countries, particularly in respect to fine-scale structures and management regulations within land-cover types (Herzog et al. 2017; Pe’er et al. 2017). Consequently, differences in historical development and agri-environmental measures resulted in regional differences of resource availability within identical land-cover types. Yet crucially, it is the availability of resources and not land cover type that drives species occurrence (Cunningham and Johnson 2006; Fuller 2012; Habel et al. 2015).

Due to their complex resource requirements and association with other taxonomic groups, farmland birds are commonly used as indicators of land-use intensity and farmland biodiversity (Gregory et al. 2005; Morelli et al. 2014). Although many birds forage in crops, most farmland birds rely on resources for breeding and roosting that are complementary to those offered in arable fields (Vickery and Arlettaz 2012). Large trees, for example, offer above-ground breeding sites, shelter and complementary food and have thus been highlighted as a crucial resource for many farmland birds (Manning et al. 2006; Bock et al. 2013). Likewise, extensively managed meadows offer ground-nesting sites, high food abundance and accessibility (Schaub et al. 2010; Ekroos et al. 2019). By altering meadow management intensity or replacing old trees, local management can modify the suitability of identical land-cover types for birds and consequently compromise the validity of proxies used by HSM. This may ultimately result in observed discrepancies between predicted suitability by HSM and occurrences of the species of concern. While the challenges of predicting habitat suitability across regions have been highlighted before (Brambilla et al. 2009; Wan et al. 2019), to the best of our knowledge, the performance of HSM to predict resources across borders has never been tested on the ground. Crucially, this issue is of general relevance for HSM based on large-scale remote sensing data but may be particularly pronounced when political borders are involved.

Here, we investigate the associations between predicted habitat suitability and resource availability across political boundaries by means of a characteristic species for extensively cultivated farmland—the little owl (*Athene noctua*). In areas of identical (i.e. highest) suitability according to a multi-level habitat suitability model (Fattebert et al. 2018) in two adjacent countries (Germany vs. Switzerland), we identify the differences in land cover, land-use intensity and resource availability. We predict land cover and land-use richness to be similar between the selected study areas, as they were underlying the development of the HSM. In contrast, we suspect differences in land-use intensity and resource availability between the two countries, due to existing differences in history, policy and socio-cultural context that are not accounted for in the modelling process. With this study we aim at assessing the performance of HSM based on geodata to predict resources across political borders to raise awareness of an important limitation of HSM, as well as assessing drivers of resource availability.

## Material and methods

### Study species and study area

The study was performed in Baden-Württemberg, south-western Germany (48.78° N, 9.18° E; Online Source Fig. S1) and the northern lowland areas of Switzerland (46.95° N, 7.44° E; Online Source Fig. S1). The study areas are characterized by a mosaic of intensively cultivated agricultural fields, sown leys, permanent meadows, vineyards and orchards (Grüebler et al. 2014; Apolloni et al. 2018). Whereas Germany and Switzerland were broadly inhabited by little owls in the middle of the last century, populations recently declined as they did across many parts of Europe with only a few breeding pairs left in Switzerland (Schaub et al. 2006; Thorup et al. 2010; Habel et al. 2015). In contrast, a vital little owl population remains in Baden-Württemberg (Bock et al. 2013; Grüebler et al. 2018). The little owl prefers widely open agricultural landscapes and is often associated with extensively cultivated standard fruit tree orchards and permanent meadows in Central Europe (Van Nieuwenhuyse et al. 2008; Šálek et al. 2016; Fattebert et al. 2018). Vertebrate (birds, small mammals, amphibians) and invertebrate (arthropods, earthworms) prey is mainly caught on bare ground or grassland with low vegetation (Grüebler et al. 2018). Little owls are cavity breeders that prefer cavities with openings of at least 6 cm diameter and 20 cm depth and multiple openings for breeding (Tomé et al. 2004; Bock et al. 2013). In addition to tree cavities, cavities in stone piles, stacks of wood, buildings or even in the ground are used (Tomé et al. 2004; Van Nieuwenhuyse et al. 2008). Likewise, roosting sites also include stacks of wood, buildings and tree crowns in addition to tree cavities (Bock et al. 2013).

### Sampling design

Using species occurrence and radio-tracking data from south-western Germany and Switzerland, Fattebert et al. (2018) developed a multi-level habitat suitability model (McGarigal et al. 2016) for the little owl. They first modelled habitat suitability at the first-, second- and third-order of selection (Meyer and Thuiller 2006) using use-available design resource selection functions (Manley et al. 2002). At each order, models were reduced from a full model containing the variables elevation, slope, NDVI, percent land cover class (cropland, forest, orchard, meadow, built-up), and distance to forest edge, using a manual backward step-wise variable selection. Habitat suitability was then mapped by projecting the RSF’s to the whole study area. External validation showed that all three models had a high predictive ability of out-of-sample validation data at their training extent, with a positive correlation between the number of validation occurrence and the value of the habitat class (Spearman rho r_s_ range = 0.886–1.000, p < 0.005; Boyce 2006). Finally, to account for conditional dependencies across scales, Fattebert et al. (2018) integrated the three order-specific models into a single map by multiplying the three layers. Importantly, the resulting all-in-one, multi-level habitat layer had a high predictive ability of out-of-sample little owl occurrence data in Germany and Switzerland (r_s_ = 0.952, p < 0.005). We now selected 99 random points in south-western Germany and Switzerland respectively, in the highest suitability class identified by the multi-level habitat model (Online Source Fig. S1). Points were centred to the next available fruit tree (moved by mean ± 1 SE: 279.6 ± 84.8 m), because fruit trees are known as the main breeding site of little owls in both regions. Around each point, a square of 1 ha (100 m × 100 m) delineated the area for sampling.

In each square (hereafter “plot”) we recorded habitat parameters related to important resources for the survival and reproduction of little owls (Table 1) between late April and early August 2013. Land cover was characterized by (1) the area covered by permanent meadows and (2) land-use richness calculated on the basis of the different arable land-cover types (Online Source Table S1). Land-use intensity was measured by (1) determining the management intensity of permanent meadows with the help of indicator plant species (Jenny et al. 2011), (2) registering the occurrence of grazing, (3) assessing if there were different meadow cutting regimes present in the same plot (i.e. a diversity of cutting dates) and (4) summing the occurrences of different small structural elements (see Table 1 for details). Finally, we assessed the availability of three crucial resources: (1) tree cavities, (2) roosting sites and (3) small rodents. Tree cavities (> 6 cm diameter and > 20 cm depth; Bock et al. 2013) were summed for the entire plot. Roosting sites were defined as open spaces in structures such as buildings, palette stacks or similar structures (but excluding nestboxes, tree crowns and tree cavities) large enough for a little owl to hide or find shelter (Bock et al. 2013). The availability of small rodents, one of the little owls’ main food source (Apolloni et al. 2018), was approximated by calculating a small rodent index based on traces counted on standardized transects (Table 1; Delattre et al. 1996; Apolloni et al. 2018). Finally the number of trees (i.e. all trees standing freely, in groups or orchards but not in hedges) was counted in each plot and used as covariate in some models (see Table 2 and statistical analyses section). In addition the diameter at breast height was recorded for every tree to investigate the tree-specific occurrence of cavities.

**Table 1:** Habitat parameters recorded for each sampling plot

Parameter name	Description	Class	Levels/range	Species needs
*Land cover*				
Permanent meadow area	Area (ha) covered by permanent meadows	Numeric	0.02–1.00	Food availability & accessibility (Šálek and Lövy 2012; Šálek et al. 2016)
Land-use richness	Number of different land-cover types (see Online Source Table S1)	Numeric	1–5	Food availability & accessibility (Van Nieuwenhuyse et al. 2008)
*Land-use intensity*				
Low-intensity meadows	Occurrence of permanent meadows of low management intensity according to Jenny et al. (2011)	Binary	0; 1	Food availability (McCracken and Tallowin 2004)
Grazing	Occurrence of grazed areas	Binary	0; 1	Food availability & accessibility (Šálek and Lövy 2012; Apolloni et al. 2018)
Different cutting regimes	Occurrence of different cutting regimes	Binary	0; 1	Food accessibility (Šálek and Lövy 2012; Vickery and Arlettaz 2012)
Small structural elements	Sum of occurrences of dry stone walls, stone piles, stacks of wood, brush piles, hedges, summer houses, unmaintained buildings and equipment shelter buildings	Numeric	1–8	Food availability, food accessibility, shelter (Van Nieuwenhuyse et al. 2008; Šálek et al. 2016)
*Resource availability*				
Tree cavity number	Total number of tree cavities > 6 cm diameter and > 20 cm depth	Numeric	0–100	Nesting places, shelter (Tomé et al. 2004; Bock et al. 2013; Habel et al. 2015)
Roosting site	Occurrence of potential little owl roosting site(s) (e.g. in buildings, palette stacks etc. excluding nestboxes, tree crowns and tree cavities)	Binary	0; 1	Shelter (Bock et al. 2013)
Small rodent index	Total predicted small rodent traces per ha: Number of runways, vole piles and holes counted on three 5 m × 1 m transects (Apolloni et al. 2018) with one each randomly placed on meadows, field margins and orchards divided by transect area and multiplied times the cover of the respective land use (meadow, field margin, orchard)	Numeric	0–11 260	Food availability (Apolloni et al. 2018; Grüebler et al. 2018)
*Covariates*				
Number of trees	Total number of trees—i.e. all trees standing freely, in groups or orchards. Trees in hedges were omitted	Numeric	1–242	–
Sampling date	The date of sampling	Date	2013/04/22–2013/08/07	–
*Individual tree traits*				
Cavity occurrence	Occurrence of a cavity > 6 cm diameter and > 20 cm depth (Bock et al. 2013) in individual trees	Binary	0; 1	–
Tree dbh	Individual tree diameter at breast height in cm	Numeric	1–118	–

All parameters were recorded for the plot area of 1 ha

**Table 2:** Model summary of habitat differences between German and Swiss plots (country) including sampling date as a covariate where assumed to be important

	Model type^a^	Estimate	95% CrI
*Land cover*			
Permanent meadow area^b^	Lm		
Country		− 0.055	− 0.115 to 0.006
Land-use richness	Poisson glm		
Country		0.100	− 0.098 to 0.297
*Land-use intensity*			
Low-intensity meadows	Binomial glm		
Country		**− 2.358**	**− 3.050** to **− 1.646**
Grazing	Binomial glm		
Country		**1.979**	**1.232** to **2.709**
Different cutting regimes	Binomial glm		
Country		**− 2.480**	**− 3.284** to **− 1.695**
Sampling date		**0.026**	**0.014** to **0.038**
Small structural elements	Poisson glm		
Country		**− 1.150**	**− 1.381** to **− 0.920**
*Resource availability*			
Tree cavity number	Negbinom glm		
Country		**− 1.760**	**− 2.089** to **− 1.424**
Roosting site	Binomial glm		
Country		**− 1.289**	**− 1.909** to **− 0.681**
Small rodent index^c^	Lm		
Country		**− 7.668**	**− 13.980** to **− 1.695**
Sampling date		**− 0.121**	**− 0.216** to **− 0.028**

Shown are the model type, parameter estimates and 95% CrI. Effects with CrI not overlapping zero are printed in bold

^a^Lm = linear model with Gaussian error distribution and identity-link function; Binomial glm = generalized linear model with binomial error distribution and logit-link function; Poisson glm = generalized linear model with poisson error distribution and log-link function; Negbinom glm = Bayesian generalized linear model with negative binomial error distribution and log-link function

^b^Arcsine-square root-transformed

^c^Square root-transformed

### Statistical analyses

Differences in habitat features between plots in south-western Germany (hereafter ‘German plots’) and Switzerland (hereafter ‘Swiss plots’) were evaluated by fitting individual models for the recorded habitat parameters (Table 2). Linear models (LMs) and generalized linear models (GLMs) were used to model habitat parameters with country (two levels: “Germany”, “Switzerland”) as main predictor, whereas the best-fitting model type and error distribution was identified based on the data collection process and investigating residuals based on model validation plots. Permanent meadow area was arcsine square root- and small rodent index square root-transformed to fulfil the normality criteria for LMs (Table 2). GLMs with negative binomial error distribution were fitted with the R package “rstanarm” (Stan Development Team 2016) in case of overdispersion for GLMs with poisson error distribution. For evaluating cutting regimes and small rodent traces, we additionally included sampling date as covariate, as mowing and small rodent availability likely fluctuate in time.

To investigate the determinants of cavity availability in more detail, we additionally grouped individual trees into dbh-classes (Online Source Table S2) and plotted the dbh class means against total cavity numbers and total number of trees per dbh-class. Furthermore, to model tree cavity occurrence probability, we fitted a generalized linear mixed-effects model (GLMM) with binary error distribution (logit-link function) for individual trees with country, standardized dbh and its second-order polynomial, as well as the two-way interactions of country with standardized dbh and its polynomial as fixed effects and plot id as random effect. One tree with a dbh of 186 cm (out of 8892 sampled trees) was excluded as it represented an obvious outlier biasing cavity-occurrence patterns (Table 1).

Bayesian posterior distributions were simulated (5000 simulations) for model estimates with the R package “arm” (Gelman and Su 2016) for LMs and GLMs and with “rstanarm” (Stan Development Team 2016) for negative binomial GLMs and used to calculate 95% credibility intervals (95% CrI) and model predictions. Effects with 95% CrI not overlapping zero were termed statistically “significant”. All statistical analyses and figures were done in R v. 3.5.2 (R Core Team 2018).

## Results

**Fig. 1:**
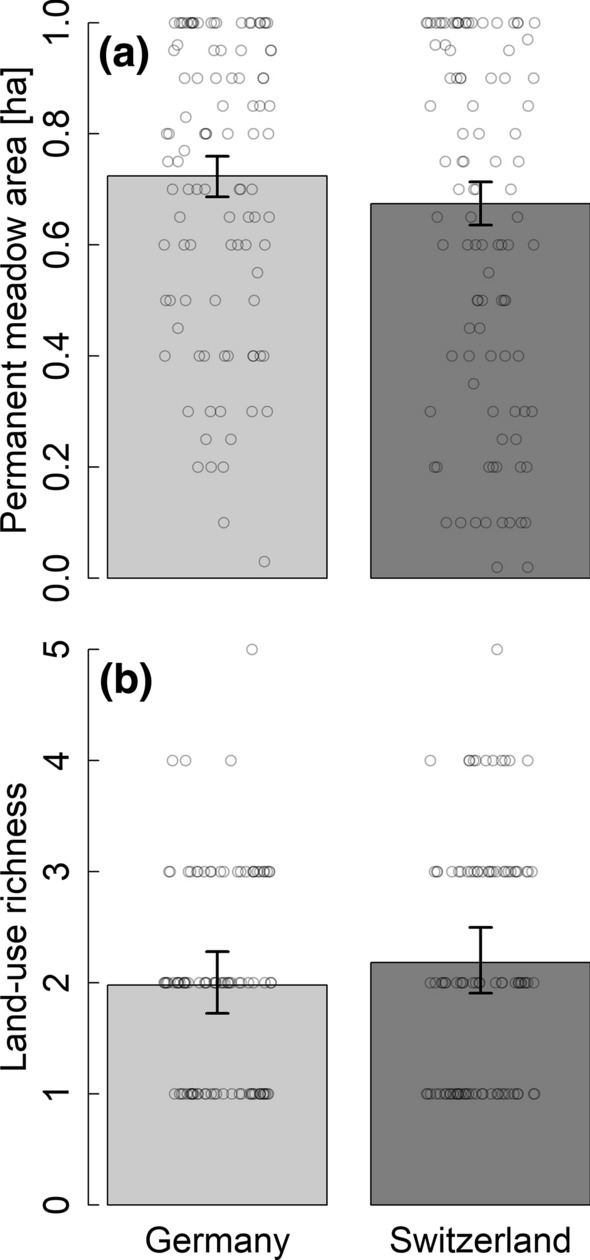
Differences in land cover between plots in high suitability areas in Germany and Switzerland. Model predictions and 95% CrI of **a** permanent meadow area and **b** land-use richness in plots in south-western Germany (light grey bars) and Switzerland (dark grey bars). Data are shown per 1 ha sampling plot. Points represent raw data

**Fig. 2:**
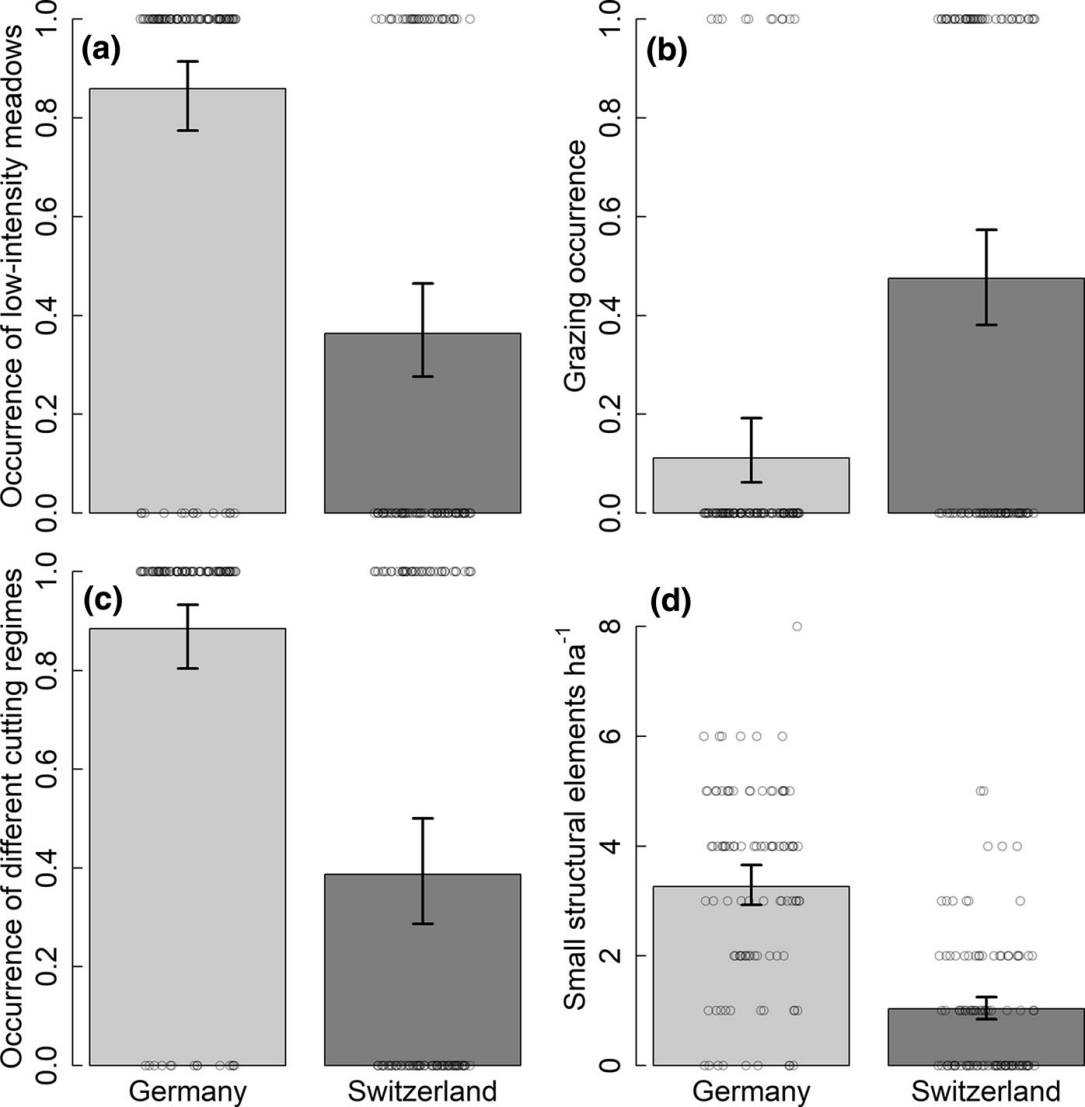
Differences in land-use intensity between plots in high suitability areas in Germany and Switzerland. Model predictions and 95% CrI of **a** occurrence of low-intensity meadows, **b** occurrence of grazed areas, **c** occurrence of different cutting regimes, and **d** small structural elements in plots in south-western Germany (light grey bars) and Switzerland (dark grey bars). Data are shown per 1 ha sampling plot. Points represent raw data

**Fig. 3:**
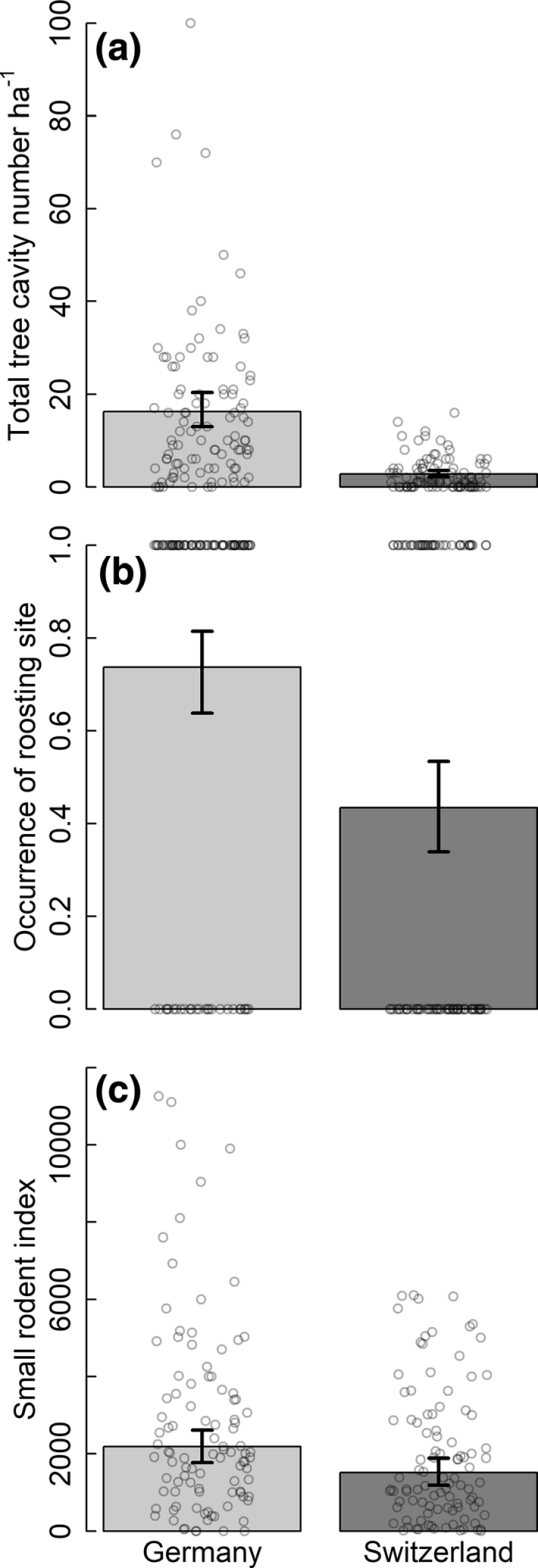
Differences in resource availability between plots in high suitability areas in Germany and Switzerland. Model predictions and 95% CrI of **a** total number of tree cavities, **b** occurrence of roosting site and **c** small rodent index in plots in south-western Germany (light grey bars) and Switzerland (dark grey bars). Data are shown per 1 ha sampling plot. Points represent raw data

There was no difference in land cover (area covered by permanent meadows and land-use richness) between plots of high habitat suitability in Germany and Switzerland (Table 2; Fig. 1). However, land-use intensity differed strongly between German and Swiss plots. Low-intensity meadows and different cutting regimes occurred more often, meadows were grazed less often, and small structural elements were more abundant in German than Swiss plots (Table 2; Fig. 2). The availability of resources such as tree cavities, roosting sites and rodents (i.e. small rodent index) was higher in German than in Swiss plots (Table 2; Fig. 3). Tree cavity number was positively correlated with tree number (Online Source Fig. S2) but the number of cavities was also higher in German than in Swiss plots when accounting for the number of trees in the model (model estimate country = − 0.757; 95% CrI = − 1.141 to − 0.372; model estimate number of trees = 0.015; 95% CrI = 0.011 to 0.020), revealing a higher number of cavities per tree in German compared to Swiss plots. As expected, the occurrence of different meadow cutting regimes and the number of expected small rodent traces varied with sampling date (Table 2).

**Fig. 4:**
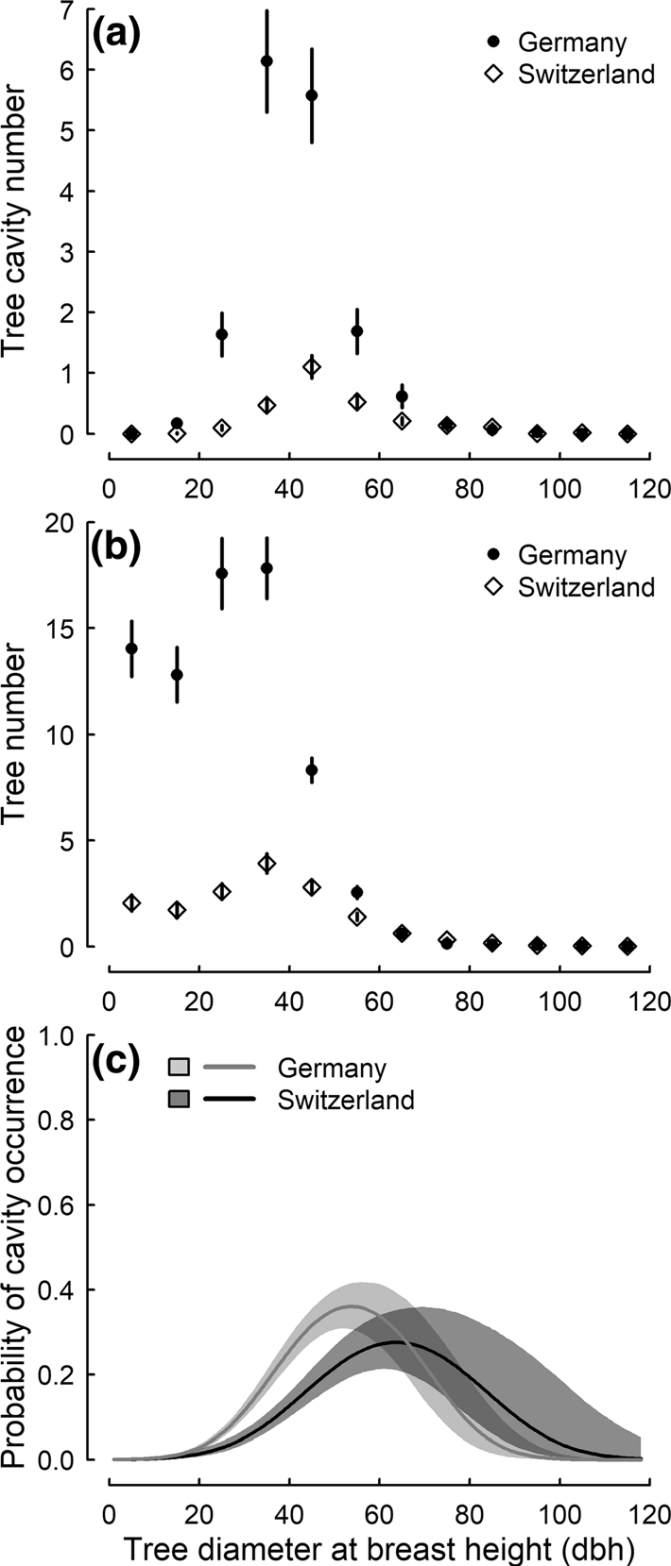
Factors underlying tree cavity availability in German plots (Germany) and Swiss plots (Switzerland). Mean ± 1 SE number of **a** observed tree cavities per 1 ha plot and **b** number of trees per 1 ha plot in different tree size classes in south-western Germany (filled circles) and Switzerland (open diamonds), and **c** model predictions and 95% CrI (shaded areas) of probability of cavity occurrence per individual tree in response to tree diameter at breast height (dbh) in plots in south-western Germany (light grey line and light grey shaded area) and Switzerland (dark grey line and dark grey shaded area)

The different number of cavities per tree might be due to differences in the size distribution of the trees. Most cavities were found in trees of a dbh between 20 and 60 cm with generally more cavities found in German than Swiss plots (Fig. 4a). Most trees had a dbh smaller than 60 cm with consistently more trees counted in German than Swiss plots up to a dbh of 60 cm (Fig. 4b). Yet, indeed the probability of a tree having a cavity followed a quadratic curve that differed between countries (Online Source Table S3; Fig. 4c). In German plots the probability for a tree having a cavity peaked at p = 0.36 for trees with a dbh of 53.9 cm, whereas in Swiss plots it peaked at p = 0.28 for trees with a dbh of 63.9 cm (Fig. 4c). While trees with a low bhd (between 20 and 60 cm) had a higher probability to contain a cavity in German compared to Swiss plots, trees with a large bhd (> 80 cm) had a higher probability for a cavity in Swiss plots (Fig. 4c).

## Discussion

We found large differences in land-use intensity and the availability of resources between plots of high predicted habitat suitability in south-western Germany and Switzerland. Land-use intensity was lower and resource availability higher in German compared to Swiss plots. These results illustrate the limitations of HSM based on land cover proxies to predict for land-use intensity and resources across borders and emphasize the necessity of specifically assessing key resources for designing effective conservation measures.

Large differences in land-use intensity and resource availability, but not land cover, corroborate our hypotheses that HSM account well for land cover but insufficiently predict differences in suitability arising due to differences in resources. While a strong correlation between land cover classification and resource availability may allow accurate predictions for suitability in proximity of its calibration, the Swiss-German border alters this association. Remarkably, the multi-level HSM for little owls was calibrated with species occurrence data from Switzerland and Germany at the population level, whereas data for calibration at the individual level (home range placement and within-home range selection) were only available from radio-tracking data collected in Germany (Fattebert et al. 2018). At the small scale, the habitat suitability model is therefore mainly influenced by the association between little owl occurrence and land cover classification in Baden-Württemberg. Crucially, our findings do not compromise HSM as an invaluable tool to detect areas with a suitable ecological infrastructure based on land-cover classification data. In contrast, measures to improve resource availability are best focused on areas of high predicted suitability. Yet, the discrepancy between HSM predictions and resource availability at a fine scale may explain the absence of little owls from areas with high suitability scores in Switzerland.

The following mechanisms can explain how differences in land-use intensity and resources affect the absence of little owls in the Swiss compared to the German study areas. High intensity of meadow management is known to reduce food abundance and accessibility to birds (McCracken and Tallowin 2004), which is reinforced by low spatio-temporal heterogeneity of management as induced by the lack of different cutting regimes (Vickery and Arlettaz 2012). In addition, the reduced occurrence of small structural elements in Swiss plots decreases structural diversity which is known to benefit birds in various ways (Van Nieuwenhuyse et al. 2008; Šálek et al. 2016). Farmsteads, for example, represent bird diversity hotspots due to their provisioning of foraging grounds, roosting and nesting sites (Grüebler et al. 2010; Hiron et al. 2013). Furthermore, intermediate grazing intensities, with a high plant diversity and heterogeneity in sward structure that encourage rich food sources and facilitate accessibility, is known to support more farmland birds than low or high grazing intensity (McCracken and Tallowin 2004; Apolloni et al. 2018). Our observations suggest that grazing intensity in our Swiss plots was high, offering low plant diversity and reduced accessibility due to dense and monotonous swards.

In addition, the three directly measured resources known to be crucial for little owls were less abundant in Swiss compared to German plots: First, the supply of suitable tree cavities that is known to limit populations of many secondary cavity breeders (Cockle et al. 2010; Habel et al. 2015). The lower density of tree cavities in Swiss compared to German plots was a combined result of lower tree density and of a lower likelihood of individual trees containing cavities. As expected, the occurrence probability of tree cavities increased with tree size (Schwarze et al. 2000; Cockle et al. 2010; Grüebler et al. 2013). However, the likelihood for cavities decreased after reaching a maximum at 53.9 cm (German plots) and 63.9 cm (Swiss plots) dbh, suggesting a selective removal of large damaged trees. Second, lower availability of roosting sites in Swiss plots may limit little owls due to their key importance as a shelter from predators, disturbance and adverse weather throughout the year (Bock et al. 2013; Grüebler et al. 2014). Finally, the lower small rodent index in Swiss compared to German plots likely represents a direct estimate of reduced food supply for little owls in Swiss compared to German plots (Grüebler et al. 2018).

A multitude of drivers can explain the differences in land-use intensity and resources between German and Swiss areas of highest habitat suitability. Historically, governments have for example supported the transformation of traditional fruit plantations (i.e. “Streuobst”; Herzog 1998) into dwarf tree orchard systems by subsidising the clearing of standard fruit trees in view of creating a more market-oriented fruit production. This development was more severe in Switzerland (minus 70% traditional orchards) compared to south-western Germany (minus 37% in Baden-Württemberg; Herzog 1998), where large “Streuobst” landscapes remain.

In addition, there are differences in policy and markets between south-western Germany and Switzerland that result in major structural differences. For example, farmland in south-western Germany is dominated by arable crops (57.9% of agricultural production area in 2016; permanent grassland = 38.5%; Statistisches Landesamt Baden-Württemberg 2019) but by grassland in Switzerland (70.3% in 2016; 26.0% arable crops; Bundesamt für Statistik 2019), and animal numbers per area are lower in south-western Germany (total 45 cows, sheep and goats per km^2^ in 2016; Statistisches Landesamt Baden-Württemberg 2019) than Switzerland (total 65 cows, sheep and goats per km^2^ in 2016; Bundesamt für Statistik 2019). These differences likely result in a higher intensification pressure in Switzerland—and particularly more intensified grassland management. In addition, differences in property rights may result in orchards or allotments being more often owned by non-farmers in south-western Germany compared to Switzerland (Bundesversammlung der Schweizerischen Eidgenossenschaft 2014; Statistisches Landesamt Baden-Württemberg 2017), which again results in lower constraints for cost-effective management in south-western Germany. The fact that tree cavity occurrence was lower in Swiss than in German plots indeed suggests that selective removal of damaged trees was more pronounced in Switzerland than in Germany or trees maintained more thoroughly—particularly for intermediate-sized trees that are used for fruit production.

To mitigate the adverse effects of agricultural intensification on biodiversity, both countries have implemented AES. Although AES on both sides of the border are highly developed and support specific measures such as extensive grassland management and traditional orchards (Baden-Württemberg: Science for Environment Policy 2017; Pe’er et al. 2017; Switzerland: Herzog et al. 2017), the Swiss scheme is more targeted to support ecological quality and measures for individual trees and structures such as flower strips, improved field margins or stone heaps (Herzog et al. 2017). However, the fact that low-intensity meadows occurred more often in German than in Swiss plots, and all investigated structures and resources were more abundant, suggests that Swiss AES measures are ineffective (see also Home et al. 2014).

We thus suppose that there are major socio-cultural drivers that contribute to the observed structural differences between Germany and Switzerland. In particular, differences in attitude, social norms and expectations may affect management intensity and resource availability. Indeed, farmer’s attitudes and subjective norms have been identified as a major factor limiting the success of Swiss AES (Home et al. 2014). Swiss farmers’ willingness to invest in conservation is often impaired by their concerns to be considered unproductive and tolerance towards non-productive land uses is usually low (Home et al. 2014).

## Conclusions

Our study raises concern that HSM based on land cover data may often fail to extrapolate suitability across political borders. This is because political borders can impact the associations between land cover classifications and resource availability. While HSM are valuable for detecting areas of potential suitability at the landscape scale, this does not automatically imply optimal fine-scale characteristics. To fully cover a species’ conservation needs and turn areas of high-potential into ‘real habitat’ therefore requires that small-scaled studies assessing key resources complement land-cover classification based HSM (cf. Brambilla et al. 2009; Rhodes et al. 2015).

Conservation measures consequently may have to put more effort into improving quality in terms of reducing agricultural land-use intensity and providing crucial resources instead of solely focusing on the availability of important land cover types. Due to strong similarities in habitat requirements with many farmland birds, measures to restore habitat for little owls will likely benefit multiple species (Fuller 2012; Zellweger-Fischer et al. 2018). Because of this general value, resource-rich structures may be promoted by governments using horizontal agri-environment schemes (AES). Specifically, AES could add or reinforce payments for extensive meadow management and traditional orchards, assure a diversity in cutting regimes and moderate grazing intensities, promote single trees (with special focus on large trees) and small structural elements. However, the mismatch between measures supported by AES and the availability of resources between Germany and Switzerland suggests that the current Swiss AES are ineffective. Increased payments for ecological quality or more result-based payments may offer one solution to improve their success (Meichtry-Stier et al. 2014; Herzon et al. 2018). Yet, as financial payments alone are often insufficient in positively affecting farmland biodiversity (Home et al. 2014), we believe that a paradigm-shift in social norms and attitudes is needed, from conceiving semi-natural structures as obstacles to productivity, towards acknowledging their functions and services—or at least towards more tolerance for landscape heterogeneity and apparent disorderliness.

## Electronic supplementary material

Below is the link to the electronic supplementary material.

Supplementary file1 (DOCX 928 kb)

## Data Availability

The datasets generated during and/or analysed during the current study are available from the corresponding author on reasonable request.
