# Supplementary material for: Political borders impact associations between habitat suitability predictions and resource availability
Source: Landsc Ecol. 2020 Sep 4;35(10):2287–300. doi: 10.1007/s10980-020-01103-8 (PMC7524687; doi:10.1007/s10980-020-01103-8)
Supplement: Supplementary file 1 — Supplementary file1 (DOCX 928 kb) [file 10980_2020_1103_MOESM1_ESM.docx]

**Electronic Supplementary Material**

*Landscape Ecology*

Political borders impact associations between habitat suitability predictions and resource availability

Matthias Tschumi^1*^, Patrick Scherler^1,2^, Julien Fattebert^1,3^, Beat Naef-Daenzer^1^, Martin U. Grüebler^1^

Affiliation of authors:

^1^Swiss Ornithological Institute, Seerose 1, CH-6204 Sempach, Switzerland

^2^Institute of Evolutionary Biology and Environmental Studies, University of Zurich, Winterthurerstrasse 190, CH-8057 Zurich, Switzerland

^3^School of Life Sciences, University of KwaZulu-Natal, Durban 4000, South Africa

*Corresponding author:

Matthias Tschumi

Swiss Ornithological Institute

Seerose 1

CH-6204 Sempach, Switzerland

Phone: +41 41 462 99 24

E-mail: matthias.tschumi@vogelwarte.ch

ORCID: 0000-0002-7991-7780

**Table S1.** Descriptive statistics of land-cover types recorded in the 1 ha study plots. Shown are arithmetic means (Mean), standard errors (SE), minima (Min) and maxima (Max) of the percentage land cover of the individual land-cover types.

| **Land-cover type** | **Mean** | **SE** | **Min** | **Max** |
| --- | --- | --- | --- | --- |
| Recently sown | 1.7 | 0.4 | 0 | 72 |
| Oilseed rape | 1.1 | 0.2 | 0 | 45 |
| Maize | 4.1 | 0.5 | 0 | 75 |
| Winter cereals | 7.4 | 0.7 | 0 | 90 |
| Spring cereals | 1.5 | 0.3 | 0 | 60 |
| Potatoes | 0.7 | 0.2 | 0 | 70 |
| Sugar beet | 0.9 | 0.3 | 0 | 70 |
| Vegetables | 1.4 | 0.3 | 0 | 70 |
| Ley | 4.5 | 0.6 | 0 | 97 |
| Permanent grassland | 70.4 | 1.2 | 2 | 100 |

**Table S2.** Grouping of trees into size classes used for investigating cavity and tree number patterns across tree size classes.

| **Class name** | **dbh range [cm]** |
| --- | --- |
| 05 | 0 ≤ dbh < 10 |
| 15 | 10 ≤ dbh < 20 |
| 25 | 20 ≤ dbh < 30 |
| 35 | 30 ≤ dbh < 40 |
| 45 | 40 ≤ dbh < 50 |
| 55 | 50 ≤ dbh < 60 |
| 65 | 60 ≤ dbh < 70 |
| 75 | 70 ≤ dbh < 80 |
| 85 | 80 ≤ dbh < 90 |
| 95 | 90 ≤ dbh < 100 |
| 105 | 100 ≤ dbh < 110 |
| 115 | 110 ≤ dbh < 120 |

**Table S3.** Model summary of the probability of tree cavity occurrence in plots in south-western Germany and Switzerland (country) and in relation to standardized diameter above breast height (bhd) of the trees. Shown are parameter estimates and 95% CrI. Effects with CrI not overlapping zero are printed in bold

|  | **Estimate** | **95% CrI** |
| --- | --- | --- |
| Probability of cavity occurrence |  |  |
| country | **0.852** | **0.415 to 1.302** |
| bhd | **2.011** | **1.510 to 2.541** |
| bhd^2 | **-0.428** | **-0.595 to -0.263** |
| country x bhd | 0.200 | -0.364 to 0.746 |
| country x bhd^2 | **-0.215** | **-0.419 to -0.006** |

**
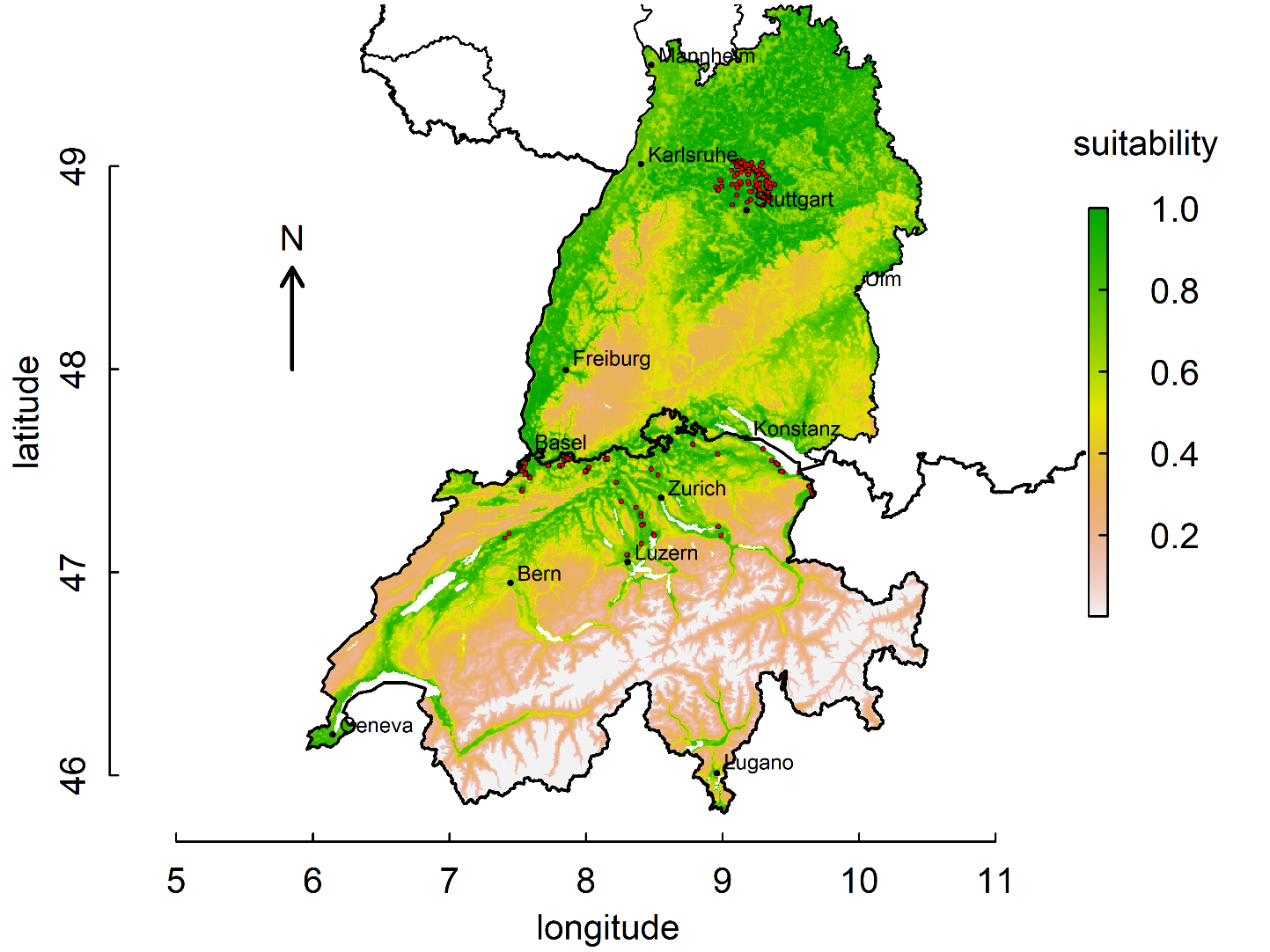
**

**Fig. S1** Map depicting Baden-Württemberg (Germany) in the north and Switzerland in the south separated by the Swiss-German border (thick black line), overlaid by the multi-level habitat suitability model values of Fattebert et al. (2018) and the study plots (red filled circles).**
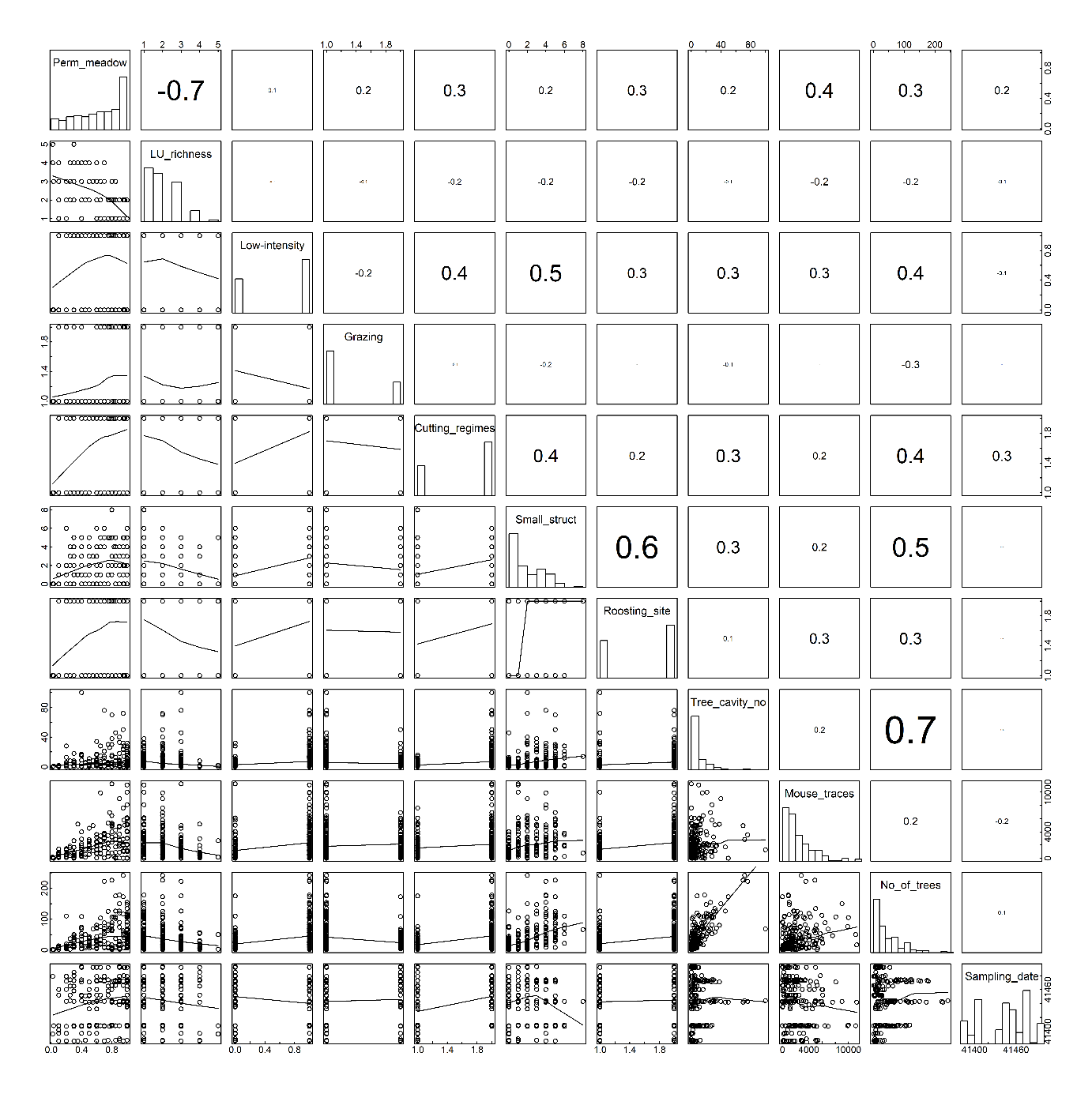
**

**Fig. S2** Pairplot of all investigate habitat parameters and covariates. The upper panel shows pair-wise correlations with font size corresponding to correlation coefficients, the diagonal panel shows histograms and the lower panel scatterplots with a LOESS smoother (Zuur et al., 2009)

**References**

Zuur, A.F., Ieno, E.N., Walker, N.J., Saveliev, A.A., Smith, G.M., 2009. Mixed Effects Models and Extensions in Ecology with R, Statistics for Biology and Health. Springer Science+Business Media LLC, New York. doi:10.1007/978-0-387-87458-6_1
